# Supplementary material for: The Systems Biology Research Tool: evolvable open-source software
Source: BMC Syst Biol. 2008 Jun 29;2:55. doi: 10.1186/1752-0509-2-55 (PMC2446383; doi:10.1186/1752-0509-2-55)
Supplement: Additional file 1 — SBRT Archive. An archive of the current version of the Systems Biology Research Tool. [file 1752-0509-2-55-S1.zip › sbrt-1.4.0/doc/developers_guide/api/sbrt/kernel/fba/proc/RandomConstraintsGeneratorV1.html]

RandomConstraintsGeneratorV1


|  |  |  |
| --- | --- | --- |
| **Overview** \| **Package** \| **Class** \| **Use** \| **Tree** \| **Deprecated** \| **Index** \| **Help** | |  |
| **PREV CLASS**   **NEXT CLASS** | **FRAMES**    **NO FRAMES**     **All Classes** |  |
| SUMMARY: NESTED \| FIELD \| CONSTR \| METHOD | DETAIL: FIELD \| CONSTR \| METHOD |  |


---


## sbrt.kernel.fba.proc Class RandomConstraintsGeneratorV1

```
java.lang.Object
  sbrt.kernel.fba.proc.FbaProcess<java.util.Map<java.lang.String,Interval>>
      sbrt.kernel.fba.proc.RandomConstraintsGeneratorV1
```

**All Implemented Interfaces:**: KernelProcess<java.util.Map<java.lang.String,Interval>>, MonitoredProcess<java.util.Map<java.lang.String,Interval>>

---

``` public final class RandomConstraintsGeneratorV1 extends FbaProcess<java.util.Map<java.lang.String,Interval>> implements MonitoredProcess<java.util.Map<java.lang.String,Interval>> ```

This class is used to generate random flux constraints.

**Author:**
:   This class was written and documented by Jeremiah Wright
    while in the Wagner lab.

---

**Constructor Summary**

`RandomConstraintsGeneratorV1(IrrevRxnFluxome fluxome)`
  
          Constructs a new random constraint generator.


| **Method Summary** | |
| --- | --- |
| `Interval` | `getActive()`             Returns the constraint used to represent an active flux. |
| `int` | `getActiveFluxes()`             Returns the number of active fluxes in each set of randomly generated flux constraints. |
| `IrrevRxnFluxome` | `getFluxome()`             Returns the fluxome for which random objective functions are to be generated. |
| `Interval` | `getInactive()`             Returns the constraint used to represent an inactive flux. |
| `int` | `getIterations()`             Returns the number of sets of random constraints to be generated. |
| `ProgressMonitor` | `getProgress()`             Returns a progress monitor for this process. |
| `void` | `run(ResultsManager<java.util.Map<java.lang.String,Interval>> resultsManager)`             Generates sets of random flux constraints and adds them to the provided results manager. |
| `void` | `setActiveFluxes(int activeFluxes)`             Sets the total number of active fluxes to be contained in each randomly generated set of flux constraints. |
| `void` | `setIterations(int iterations)`             Sets the number of sets of random flux constraints to be produced. |
| `void` | `setOff(Interval inactive)`             Sets the constraint used to represent an inactive flux. |
| `void` | `setOn(Interval active)`             Sets the constraint used to represent an active flux. |
| `void` | `setRandom(java.util.Random random)`             Sets the random number generator to the one provided. |
| `void` | `setReactions(java.util.Set<java.lang.String> reactionNames)`             Sets the reaction names for which random constraints are to be generated. |

**Methods inherited from class sbrt.kernel.fba.proc.FbaProcess**

`toString`

**Methods inherited from class java.lang.Object**

`clone, equals, finalize, getClass, hashCode, notify, notifyAll, wait, wait, wait`

**Constructor Detail**

### RandomConstraintsGeneratorV1

```
public RandomConstraintsGeneratorV1(IrrevRxnFluxome fluxome)
```

:   Constructs a new random constraint generator.

    **Parameters:**: `fluxome` - the fluxome for which random flux constraints are to be generated.


**Method Detail**

### getFluxome

```
public IrrevRxnFluxome getFluxome()
```

:   Returns the fluxome for which random objective functions are to be
    generated.

    :   **Specified by:**: `getFluxome` in class `FbaProcess<java.util.Map<java.lang.String,Interval>>`
    :   **Returns:**: the fluxome for which random objective functions are to be generated.

---


### getIterations

```
public int getIterations()
```

:   Returns the number of sets of random constraints to be generated.

    :   **Returns:**: the number of sets of random constraints to be generated.

---


### setIterations

```
public void setIterations(int iterations)
```

:   Sets the number of sets of random flux constraints to be produced.

    :   **Parameters:**: `iterations` - the number of random flux constraints to be produced.

---


### getActiveFluxes

```
public int getActiveFluxes()
```

:   Returns the number of active fluxes in each set of randomly generated
    flux constraints.

    :   **Returns:**: the number of active fluxes in each set of randomly generated flux constraints.

---


### setActiveFluxes

```
public void setActiveFluxes(int activeFluxes)
```

:   Sets the total number of active fluxes to be contained in each randomly
    generated set of flux constraints.

    :   **Parameters:**: `activeFluxes` - the total number of active fluxes to be contained in each randomly generated set of flux constraints.

---


### setReactions

```
public void setReactions(java.util.Set<java.lang.String> reactionNames)
```

:   Sets the reaction names for which random constraints are to be generated.

    :   **Parameters:**: `reactionNames` - the reaction names for which random constraints are to be generated.

---


### setRandom

```
public void setRandom(java.util.Random random)
```

:   Sets the random number generator to the one provided.

    :   **Parameters:**: `random` - the random number generator to use.

---


### getActive

```
public Interval getActive()
```

:   Returns the constraint used to represent an active flux.

    :   **Returns:**: the constraint used to represent an active flux.

---


### getInactive

```
public Interval getInactive()
```

:   Returns the constraint used to represent an inactive flux.

    :   **Returns:**: the constraint used to represent an inactive flux.

---


### setOn

```
public void setOn(Interval active)
```

:   Sets the constraint used to represent an active flux.

    :   **Parameters:**: `active` - the constraint used to represent an active flux.

---


### setOff

```
public void setOff(Interval inactive)
```

:   Sets the constraint used to represent an inactive flux.

    :   **Parameters:**: `inactive` - the constraint used to represent an inactive flux.

---


### run

```
public void run(ResultsManager<java.util.Map<java.lang.String,Interval>> resultsManager)
```

:   Generates sets of random flux constraints and adds them to the provided
    results manager. Each set of random constraints contains a constant
    number of active and inactive constraints. It's the assignment of these
    constraints to reaction names that is random.

    :   **Specified by:**: `run` in interface `KernelProcess<java.util.Map<java.lang.String,Interval>>`
    :   **Parameters:**: `resultsManager` - the results manager to which reaction names mapped to their randomly assigned constraint is added.

---


### getProgress

```
public ProgressMonitor getProgress()
```

:   Returns a progress monitor for this process.

    :   **Specified by:**: `getProgress` in interface `MonitoredProcess<java.util.Map<java.lang.String,Interval>>`
    :   **Returns:**: a progress monitor for this process.


---


|  |  |  |
| --- | --- | --- |
| **Overview** \| **Package** \| **Class** \| **Use** \| **Tree** \| **Deprecated** \| **Index** \| **Help** | |  |
| **PREV CLASS**   **NEXT CLASS** | **FRAMES**    **NO FRAMES**     **All Classes** |  |
| SUMMARY: NESTED \| FIELD \| CONSTR \| METHOD | DETAIL: FIELD \| CONSTR \| METHOD |  |


---
